# Redox Properties, Bioactivity and Health Effects of Indicaxanthin, a Bioavailable Phytochemical from *Opuntia ficus indica*, L.: A Critical Review of Accumulated Evidence and Perspectives

**DOI:** 10.3390/antiox11122364

**Published:** 2022-11-29

**Authors:** Alessandro Attanzio, Ignazio Restivo, Marco Tutone, Luisa Tesoriere, Mario Allegra, Maria A. Livrea

**Affiliations:** Dipartimento di Scienze e Tecnologie Biologiche, Chimiche e Farmaceutiche, Università di Palermo, Via Archirafi 28, 90123 Palermo, Italy

**Keywords:** betalains, human bioavailability, antioxidative, pro-oxidant activity, inflammation, dysmetabolism, cancer

## Abstract

Phytochemicals from plant foods are considered essential to human health. Known for their role in the adaptation of plants to their environment, these compounds can induce adaptive responses in cells, many of which are directed at maintaining the redox tone. Indicaxanthin is a long-known betalain pigment found in the genus *Opuntia* of cactus pear and highly concentrated in the edible fruits of *O. ficus indica*, L. whose bioactivity has been overlooked until recently. This review summarizes studies conducted so far in vitro and in vivo, most of which have been performed in our laboratory. The chemical and physicochemical characteristics of Indicaxanthin are reflected in the molecule’s reducing properties and antioxidant effects and help explain its ability to interact with membranes, modulate redox-regulated cellular pathways, and possibly bind to protein molecules. Measurement of bioavailability in volunteers has been key to exploring its bioactivity; amounts consistent with dietary intake, or plasma concentration after dietary consumption of cactus pear fruit, have been used in experimental setups mimicking physiological or pathophysiological conditions, in cells and in animals, finally suggesting pharmacological potential and relevance of Indicaxanthin as a nutraceutical. In reporting experimental results, this review also aimed to raise questions and seek insights for further basic research and health promotion applications.

## 1. Introduction

As secondary plant metabolites, phytochemicals are synthetized in response to environmental adverse conditions, or other exogenous stressors promoting an increase of reactive oxygen species (ROS), and then a change of the redox status of the plant. These molecules, which are added to the powerful antioxidant machine that includes enzymes, redox proteins, and low molecular weight molecules such as antioxidant vitamins, contribute greatly to the maintenance of the plant redox homeostasis [1,2,3]. In keeping with this knowledge and with the current advancements in the comprehension of the human redox biology [4], the health effects and mechanism of action of phytochemicals found in our daily diet, are receiving increasing attention. Despite low bioavailability and low concentration in blood and peripheral tissues, most dietary phytochemicals ultimately play an essential anti-oxidative role in animal cells. According to the concept of hormesis, these molecules, which represent an adaptive response of plants to stressful and pro-oxidant conditions [2,5], in turn behave as xenobiotics that provoke repair responses directed to maintain the redox tone of cells [6,7,8,9], making the organism that consumes them stronger than it was previously.

In a complementary, but distinct way, the ability of the organism to cope with and exploit the potential of phytochemicals may fall under the concept of Adaptive Homeostasis [10]. According to the latter, physiological cellular homeostasis is a dynamic process resulting from a continuous and transient adaptation to mild stress conditions that stimulate cytoprotective responses, sensitizing the organism to further stress, without any initial noxious stimulus and, therefore, without any repair process. Phytochemicals can elicit beneficial cellular effects by influencing redox-controlled signaling pathways that modulate the activation of transcription factors and target genes involved in phase II detoxification processes, maintenance of the cellular redox environment, and other types of cellular defences [6,7,11]. The epigenetic modulation of protein expression has also emerged as a finely tuned means to explain a life-long protection by these compounds [12]. The activity and effects of phytochemicals may be expected to change when cells are stressed in any way, or transformed, i.e., when alteration of the homeostasis, with activation/deactivation of redox-sensitive signaling mechanisms beyond the point of recovery, requires specific and diversified actions. Revealing mechanisms of cytoprotective response of specific dietary phytochemicals may serve to design potentially pharmacologically active molecules.

## 2. The Betalain Indicaxanthin

**Figure 1:**
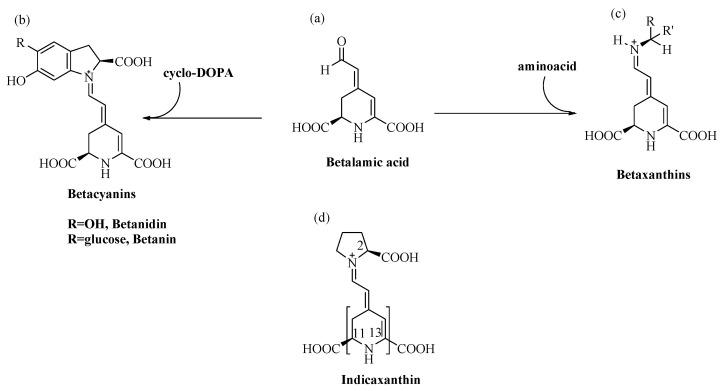
Structure of (**a**) Betalamic acid, (**b**) Betacyanins, (**c**) Betaxanthins and (**d**) Indicaxanthin (in brackets, parts with lipophilic character).

Betalain pigments are water-soluble, cationized, nitrogen-containing molecules the colours of which range from the yellow betaxanthins to the violet-red betacyanins. Specifically, the conjugation of betalamic acid with cyclo-dopa, or its glucosyl derivatives, leads to the synthesis of betacyanins (λ_max_ = 540 nm), whereas its condensation with amino acids, or the corresponding amines, leads to betaxanthins (λ_max_ = 480 nm) [13] (Figure 1).

Two of these pigments, red Betanin (betanidin 5-O-β-glucoside) and yellow Indicaxanthin (proline betaxanthin), characterize the fruits of cactus pear *Opuntia ficus indica*, L. a plant that grows in many harsh habitats of the world, from hot and arid areas of Mexico, to certain zones of Africa, Asia and the Mediterranean basin [14]. Like the much more widely spread polyphenols, secondary metabolites such as betalains address specific defence needs and represent an adaptive response of plants to which they belong. In keeping with this meaning, cactus pear fruits from plants growing wild in Sicily (Italy) show a remarkably higher content of betalains than fruits from species cultivated in the same area [15]. Betalains are mutually exclusive with anthocyanins, whose function as UV-protecting compounds they vicariate [16], which suggests their involvement against abiotic stress-generated ROS [17]. In line with these observations, berries of *Rivina humilis*, L. that synthesize betalains including Indicaxanthin [18,19], show decreased expression of superoxide dismutase (SOD) and catalase genes during development, in parallel with the accumulation of these pigments [20]. Numerous transcription factors control the expression of genes for proteins, SOD included, that confer stress resistance to plants [21]. Regulation of the activity of some as-yet unidentified factor of *Rivina humilis* by betalains is a rational hypothesis to be tested. Cell signaling indeed integrates independent stimuli into a logical functional network; cross-talk between the pigment biosynthetic pathway and pathway(s) leading to activation of stress-related genes could occur during *Rivina humilis* berry development, as a result of abiotic stress and to cope with it.

Among food-relevant betalains, redox chemistry, bioactivity and effects of Betanin, have long been studied [22], since this betacyanin is highly concentrated in the beetroot, a species widely spread and consumed all over the world. Conversely, Indicaxanthin, whose presence in edible plant sources is limited to cactus pear, and to some berries (*Rivina humilis*, L.; *Myrtillocactus geometrizans*), has long been neglected, although it was the first betaxanthin whose structure was identified. Extraction from natural sources as well as synthetic methodologies to obtain Indicaxanthin in solution have been reported since 1972 [23]; see Allegra et al. for a recent review [24]. Most of the studies encompassing physicochemical aspects, bioactivity and effects of Indicaxanthin in vitro and in vivo, have been performed by our research group in the past two decades or so.

Indicaxanthin possesses reducing properties. Antiradical and antioxidant activity emerged in solution and in cells under certain conditions [25,26,27,28,29,30]. Not less important are the physicochemical properties, with a rather large non-polar surface area (Section 3.2) that facilitate high intestinal absorption across paracellular junctions of intestinal epithelial cells [31], incorporation into cells [28,29,32,33,34,35], crossing the Brain Blood Barrier (BBB), accumulation in rat brain [36,37], and favour interactions with membrane components such as to induce cell responses [32,33,36]. Overall, many of the effects of Indicaxanthin in healthy cells, submitted to insult to simulate specific oxidative stress-linked physio-pathological conditions [32,33,35,38,39,40], have appeared as the result of restoration of cell redox homeostasis. Interestingly, an adaptive response consequent to a pro-oxidant activity has also been documented [41]. By contrast, the redox balance of some tumour cells was not modified by Indicaxanthin under the conditions applied [42,43], although the phytochemical showed anti-proliferative activity and caused apoptosis [42,43,44]. Effects observed in cellular physiology experiments with organ bath [34,45] and electro-physiological procedures [36], as well as epigenetic studies [46], suggest specific interactions with proteins. Relevantly, data from investigations in *Caenorhabditis elegans* [47,48] showed modulation of pathways that control health and longevity. Finally, findings in animal models of inflammation [49], cancer [44], obesity-related dysmetabolic conditions [50] and neuronal excitability [36], propose Indicaxanthin as a nutraceutical. Data and experimental results summarized in this review highlight key points while at the same time raising questions that open prospects for further research.

The bioavailability of Indicaxanthin from the cactus pear fruit in volunteers was measured for the first time in our lab [31]. In addition to supporting a potential nutraceutical relevance of Indicaxanthin in the human diet [51], these measurements allowed us to assess its bioactivity both in vitro and in vivo using appropriate settings that mimic human plasma concentrations after dietary ingestion, or conversely to be aware of the gap from physiological conditions.

## 3. Redox Chemistry and Physicochemical Characteristics of Indicaxanthin

### 3.1. Redox Chemistry of Indicaxanthin in Solution

Betalains are considered cationized antioxidants [52]. It is accepted that their reducing properties are tightly linked to the antiradical capacity of a cycled amine group of the structural unit, betalamic acid [53] (Figure 1). Condensation with either cyclo-DOPA or amino acid/amine to form betacyanins or betaxanthins, respectively, adds a nitrogen positively charged to the polyene system, which may limit the reactivity of these molecules. The presence of –OH groups of glucosyl derivates of cyclo-DOPA may confer additional reactivity to betacyanins (Figure 1) [54,55]. In contrast, the electron abstracted from betaxanthins could only be from conjugated π orbitals, which is hindered by the positive charge of the N-atom. Antiradical activity of Indicaxanthin evaluated in solution, was first reported in our studies and recently by other Authors [30,48,56]. Using computational chemistry methods to study the antiradical activity of various betalains, Indicaxanthin and Betanidin, a non-glycosylated betacyanin, were found to be the most active [57]. Expressed as Trolox Equivalents (TE, mol Trolox/mol Indicaxanthin), a dose-dependent scavenging ability was observed against the radical cation from 2,2′-azinobis-(3-ethylbenzothiazoline-6-sulfonic acid) (ABTS^+^), evaluated as 1.76 TE [56], and 4 TE [48]. Values of 5 TE [30] and 6 TE [48] were reported against peroxyl radicals generated by thermal decomposition of 2,2′-Azobis (2-methylpropionamidine) dihydrochloride (AAPH) in the ORAC assay. Very low TE values have been measured against 2,2′-diphenyl-1-picrylhydrazyl (DPPH.) radical (0.06 TE) and against the nitroprusside-generated NO (0.28 TE) [30].

Voltammetric potential data provided further insights on the reducing/antioxidant properties of Indicaxanthin, prompting studies on the bioactivity of the phytochemical in solution, cells and whole organism. Two characteristic peaks at 611 mV and 895 mV emerged from the differential pulse voltammogram [56], suggesting reducing capacity versus organic radicals frequently formed in cell environments [58], from alkyl-peroxyl and lipoperoxyl radicals, to hypervalent iron reactive species from heme-proteins. Indicaxanthin reduced lipoperoxyl radicals from linoleic acid methyl ester under oxidation by the 2,2′-azobis(2,4-dimethylvaleronitrile) (AMVN) azo-initiator, behaving as a classical chain breaking antioxidant, with effectiveness comparable to vitamin E [25]; the pigment was also a good one-electron-donor reductant of the hypervalent-iron oxoferryl heme of perferryl-Hb [29] and of the Compound -I and -II in the catalytic cycle of myeloperoxidase [26] (Table 1).

### 3.2. Physicochemical Properties and Interactions of Indicaxanthin with Lipid Systems

Besides the positive charge in proximity of the N1 nitrogen, Indicaxanthin possesses a number of ionizable carboxyl groups (Figure 1). Computational analysis evaluated dissociation constants, as well as molecular descriptors such as the octanol/water and octanol/buffer pH 6.0 partition coefficient (LogP, LogD), polar and non-polar surface area (PSA, NPSA) [60] (Table 2).

**Figure 2:**
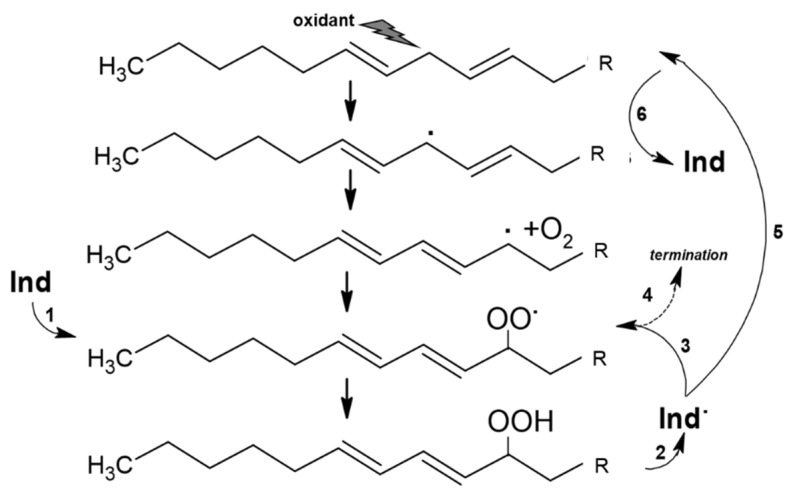
Reaction of Indicaxanthin (Ind) with lipoperoxyl radicals and possible re-cycling of Indicaxanthin short-lived radical intermediate (Ind). Ind reduces a lipoperoxyl radical (1) generating a stable lipid hydroperoxide and Ind. (2). The latter can react with a second lipoperoxyl radical (3) in a termination reaction (4) or can be reduced by reaction with polyunsatured fatty acid (5) to regenerate Ind and a lipid carbon centered radical (6) in a pro-oxidation reaction.

These measurements show that Indicaxanthin occurs as a bis-anion in a large range of pH, including physiological pH and the pH gradient at the intestine during digestion (pH 6.0–7.4) [61,62]. The molecule possesses a quite large non-polar surface (>50% surface area), which gives it a moderately nonpolar, i.e., amphipathic, character. In accordance, spectrophotometric and kinetic studies [63,64] showed that Indicaxanthin partitions in dipalmitoyl-phosphatidyl choline (DPPC, binding constant 3 × 10^3^ M^−1^, 37 °C), but not shorter dimiristoyl-phosphatidyl choline (DMPC) phospholipid bilayers. Indicaxanthin partitions at the interface between the hydrophilic head groups of DPPC bilayer and the hydrophobic core (palisade domain), an ideal solubilization site for molecules sharing hydrophilic and lipophilic moieties as well. At this level the active cyclic amine group of Indicaxanthin is in a position to eventually react with aqueous peroxyl radicals adsorbed onto the vesicular surface [65] as well as chain-propagating lipoperoxyl radicals, floating to the polar interface of the bilayer [66]. Indeed, incorporated in an aqueous dispersion of either saturated DPPC, or unsaturated soybean PC, unilamellar liposomes, Indicaxanthin reacted with peroxyl radicals from the water-soluble azo-initiator AAPH, or with lipoperoxyl radicals from the soybean PC bilayer, respectively [25]. It deserves to be mentioned that, in common with other natural antioxidants, including vitamin A, E, carotene, and flavonoids of green tea [67,68,69,70,71,72], Indicaxanthin may exhibit a pro-oxidant behavior. Compared with in-solution measurements, kinetic parameters of radical-scavenging activity and consumption kinetics of the molecule incorporated in soybean PC liposomes subjected to oxidation, revealed a partial loss of efficiency, in terms of inhibition period and kinetic chain length, critically dependent on concentration and ratio to unsaturated lipids. Re-cycling of a Indicaxanthin short-lived radical intermediate, at the expenses of PUFA, with generation of a novel lipoperoxyl radical and oxidation chain, has been suggested to rationalize this behavior [25] (Figure 2). However, under the conditions applied (PC = 10 mM; peroxidation rate, Rp = 0.25 × 10^6^ Ms^−1^), actual pro-oxidant effects were not observed until an Indicaxanthin:PUFA molar ratio of 1:500.

The amphiphilicity of Indicaxanthin allows its binding to low density lipoproteins (LDL). Ex vivo experiments [27] showed a saturable binding after spiking of human plasma with amounts of Indicaxanthin from 5 to 100 μM, with a maximum of 0.52 ± 0.08 nmol per mg LDL ApoB-100. The Indicaxanthin-enriched particles were remarkably more resistant to copper-induced oxidation than homologous control LDLs [27]. Consistently, a human dietary approach [31] demonstrated that 0.098 ± 0.012 nmol Indicaxanthin per mg Apo B-100 (49 ± 8 milli mol per mol Apo B-100) occurred in the LDLs isolated three hours after ingestion of cactus pear fruit pulp, when the peak plasma of the pigment was around 7 μM. Assuming a 1:1 molar ratio of ApoB-100 to LDL, a molecular weight of 500,000 for ApoB-100, and a 1.2 μmol/L plasma concentration of LDLs [73], the LDL-bound Indicaxanthin accounts for 0.62% of its plasma content—a percent distribution comparable with that observed ex vivo [27]. On this evidence, a protection by the dietary Indicaxanthin from the fruit [31] may also be expected.

Data obtained with biomimetic membranes [25,63,64] provide clear evidence for the ability of Indicaxanthin to interact with phospholipids. Biological membranes, however, possess a wide repertoire of phospholipid species, with different acyl chain length and saturation degree and with differences between cells, and between cell plasma membrane and organelles; moreover the membrane is compartmentalized in microdomains, with specific functions to control the activity of proteins involved in signal transduction; finally, composition and structure of membranes may change under pathophysiological conditions, and in particular in transformed tumor cells [74]. As a consequence of this complexity, one might expect that a phytochemical if it interacts with cell membranes could, from these interactions, modulate many different processes, such as enzymatic activities, receptor-ligand binding, cell signalling through oxidized membrane lipid components and their soluble derivatives, and transport of molecules and/or ions. Functional interactions with vitamin E, the major lipid antioxidant in cell membranes [75] have been shown [25,27,29], and interactions with membrane enzymes may play a role in the Indicaxanthin bioactivity [32,33,34] (Section 6). Thanks to its amphiphilicity, the molecule can cross cell membranes and enters cells. Indicaxanthin was recovered in the soluble fraction of red blood cells, after spiking of human plasma [29]; likewise, uptake was observed after incubation with intestinal-like differentiated Caco-2 cells [32,59]. In addition, Indicaxanthin entered the cells of mouse ileum organ preparations [34,45], of *C. elegans* [47,48] and, remarkably, was distributed in several specific areas of rat brain after oral administration of amounts consistent with diet [37].

## 4. Binding of Indicaxanthin to Proteins: In Silico Molecular Modeling and Test Tube Experiments

The discovery of biological targets for Indicaxanthin may provide indication on how the molecule can affect cellular processes. Interactions of Indicaxanthin with one phosphodiesterase (PDE) isoform were first hypothesized by studies that investigated the mechanism of action of its inhibitory effects on mouse ileal contractility [34,45] (Section 6.2). More recently, binding of Indicaxanthin to PDE-4, a isoform that is found in the ileal smooth muscle [76,77] has been predicted by an innovative molecular modelling approach that used combined reverse pharmacophore mapping, reverse docking, and text-based database search [78]. The same study reported qualitative and quantitative information about dynamic interactions of Indicaxanthin at the binding site of other proteins, namely Inositol Trisphosphate 3-Kinase (ITP3K-A), Glutamate carboxypeptidase II (GCPII), Leukotriene-A4 hydrolase (LTA4H), Phosphoserine phosphatase (HPSP), AMPA receptor (GluA3 and GluA2 subunits) and Kainate receptor (GluK1 isoform) [78]. Other computer-aided modelling approaches described the binding between Indicaxanthin and (i) the DNA methyl transferase 1 (DNMT1) [46], an enzyme involved in regulating epigenetic modifications; (ii) the N-methyl-D-aspartate receptor (NMDAR) [36], the most represented of the glutamate receptor family in hippocampus; (iii) the active form of IKKβ [79], the enzyme that controls the activity of the NF-κB inhibitor in physiological processes, as well as in inflammation-related disease and cancer [80]. Chemical details of the in silico methods and interactions of Indicaxanthin with proteins are described elsewhere [24,36,61].

Through modulating the activity of key enzymes and receptors, Indicaxanthin could influence the course of many physiological and pathophysiological processes. As reported in the following sections, Indicaxanthin can indeed influence inflammatory pathways, counteract cancer development, and appears as a potential natural neuro-modulating agent. To what extent direct interactions with specific proteins are involved in the observed effects cannot be decided before assays with purified enzymes and factors are performed, to assess if the ideal kinetics can approach the conditions in cells and tissues. At any instance, the in silico evidences may prompt further studies to test Indicaxanthin as a potential lead molecule for the prevention or the treatment of various disorders.

Direct interactions of Indicaxanthin with enzyme proteins emerged from test-tube experiments. Gomez-Marques et al. [30] showed that 160 μM Indicaxanthin inhibited the activity of purified α-amylase and hyaluronidase by 98% and of α-glucosidase by over 88%, under the reported experimental conditions. The inhibition of α-amylase and α-glucosidase in the intestinal carbohydrate digestion may be important for potential management of hyperglycemia-linked type 2 diabetes by natural and possibly dietary-related compounds [81,82]. In addition, inhibiting hyaluronidase may be convenient in chronic inflammatory conditions, e.g., inflammatory joint disease, osteoarthritis and skin aging [83], as well as to control steps of the wound healing process [84]. Further studies in cell environments or in in vivo models of pathology, with amounts of Indicaxanthin compatible with dietary intake [31,56], could support the suggested beneficial effects through inhibition of these enzymes.

## 5. Bioavailability of Indicaxanthin

Indicaxanthin has been shown to have higher bioavailability than most phytochemicals [85,86], including other betalains [31,52,62], which appeared the result of several factors. Studies with volunteers who received 500 g cactus pear fruit pulp accounting for 28 mg Indicaxanthin, revealed a high absorption of the molecule, with a plasma peak of 6.9 ± 0.54 μM at 180 min after ingestion (Tmax), with a T1/2 of 156 min, and urinary excretion of 76% after 12 h [31]. This study also revealed that Indicaxanthin enters the bloodstream in its native form, indicating solubility and absence of metabolic transformation in enterocytes or hepatocytes. While contributing to high bioavailability, the absence of glucuronidation, sulfation or methylation may be advantageous for eventual interaction with membranes and/or membrane effectors at the target tissues. Functional and mechanistic data relevant to digestion and absorption of Indicaxanthin, achieved in vitro by a simulated step-by-step digestion process [87] and in human Caco-2 cells treated to mimic normal enterocytes [60], were consistent and justified the bioavailability measured in vivo [31]. According to these investigations either dietary Indicaxanthin from cactus pear fruit pulp, or even the purified compound, possess high digestive stability, with a minor 25% loss at the gastric pH only, and the remaining totally recovered after the intestinal-like step in the soluble fraction, potentially available for the absorptive epithelial cells [87]. Recent research substantially confirms the high digestive stability and bioaccessibility of Indicaxanthin from cactus pear fruits [88]. On the other hand, other in vitro digestion studies report bioaccessibility around 16% for Indicaxanthin from the berry *Myrtillocactus geometrizans* [89], possibly indicating that the food matrix had strongly influenced the digestion process.

Kinetics of the trans-epithelial transport of Indicaxanthin accross intestinal cells were investigated in differentiated Caco-2 cell monolayers by the aid of Transwell^R^ inserts. Molecular mass and physico-chemical characteristics (electric charge, lipophilicity, Section 3.2) appeared to be essential to the transport, by paracellular diffusion, with a high permeability coefficient (P = 4.4 × 10^−6^ cm s^−1^), at pH 6.0, in a concentration-dependent mode, without involvement of H^+^-dependent carriers, nor efflux transporters [60]. Other studies report a lower coefficient (P = 8.9 × 10^−7^ cm s^−1^) at pH 6.5 [59]. Overall, the molecule’s high resistance to the digestive steps, and easy of diffusion at the intestinal epithelium account for the high bioavailability of Indicaxanthin from fruits of cactus pear [31]. Other pharmacokinetic measurements in rats receiving pure Indicaxanthin in a single oral dose of 2 μmol/kg body weight, which is consistent with a reasonable dietary amount from cactus pear fruit, confirmed a plasma peak of the molecule at 2 h after administration, with a total amount excreted of 21% of the dose ingested [49]. Differences in gastrointestinal systems and different digestive degradation and absorption could explain the results obtained in rats compared with human studies [31].

Noticeably, the dietary Indicaxanthin may have a direct action at the intestine, where its concentrations can be quite higher than in blood. Since Indicaxanthin does not appear to be modified by intestinal enzymes [31], experimental set-ups to study potentially protective effects for human intestinal cells, could take into account amounts of Indicaxanthin well above those measured in plasma.

## 6. Modes of Action of Indicaxanthin in Cells and Tissues

In vitro evidence to date in cells and tissues does not provide a unified picture of the molecular mechanisms mediating the action of Indicaxanthin; rather different mechanisms have emerged in relation to different stimuli and conditions in both healthy and transformed cells. Many of the activities appear to be geared toward restoring cellular redox homeostasis, correcting dysfunction generated by oxidative stress, and modulating signaling pathways that control vital processes in healthy cells [90]; other activities, apparently independent of cellular redox balance, have also been observed [34,36,45].

### 6.1. Indicaxanthin, Redox Balance and Oxidative Stress in Healthy Cells

Phytochemicals are recognized as valuable in maintaining cellular redox homeostasis. To what extent is the redox chemistry of Indicaxanthin involved in its effects on cells?

Data so far show that Indicaxanthin is stable and does not interfere with the physiological redox state of healthy cells, in the absence of pro-oxidant stimuli. This was observed with various cell lines incubated with amounts in a fairly wide range of concentrations, including postprandial blood levels (5–10 μM) up to more than one order of magnitude in excess, for time intervals from 6 h to 72 h [33,38,39,41,42,44]. On the other hand, the extent and nature of the pro-oxidant insult has appeared to be decisive for its response and activity.

#### 6.1.1. Indicaxanthin between Antioxidant vs. Pro-Oxidant Activity

Incorporated ex vivo in human red blood cells, both normal or from thalassemia patients, after incubation of plasma with concentrations of nutritional interest (1–10 μM), Indicaxanthin prevented the oxidation of membrane lipids promoted by the stable organic oxidant cumene hydroperoxide (CumOOH), and delayed the oxidative hemolysis, being totally consumed [28,29]. Indicaxanthin did not have any effect in the absence of CumOOH.

In contrast, Indicaxanthin at 100 μM acted as a pro-oxidant in an experimental set-up in which murine macrophages were exposed to the bacterial lipopolysaccharide (LPS) [41]. In this inflammation model, the activation of TLR-4 receptors and early ROS generation through NOX activity or mitochondria [91,92,93] cause oxidation of membrane lipids. Indicaxanthin promoted a significant and time-dependent elevation of lipoperoxides that ultimately resulted in the formation of the lipid mediator hydroxynonenal (HNE), an end-product of the oxidation of arachidonic acid. Known to start hormetic and adaptive responses [94,95,96,97], HNE may promote the expression of enzymes involved in the eicosanoid biosynthesis [98,99], with release of pro-resolution factors that bring inflammation to an end [100,101]. In essence, Indicaxanthin modulated the inflammatory response of macrophages by directing the biosynthetic pathway toward the synthesis of the anti-inflammatory proresolvin cyclopentenone [41]. The prooxidant activity and relevant production of cyclopentenone were positively related with concentrations as high as 50 and 100 μM, and were completely counteracted by equivalent amounts of the lipoperoxyl radical-scavenger vitamin E; no effect was observed in the absence of LPS, whatever the Indicaxanthin concentration. In consistency with the activity of high concentrations of Indicaxanthin in peroxidizing liposomes [25], these results provide evidence that recycling of an active Indicaxanthin intermediate at the expense of PUFAs can also occur in a cellular membrane.

The unconventional anti-inflammatory effect of Indicaxanthin generated through HNE can fall in the frame of the ability of oxidants to modulate cell signaling, and indicate that beneficial effects may be expected from, and in fact are often due to pro-oxidant activities of natural compounds [102]. In this context, hormetic effects and adaptive responses through pro-oxidant activity of polyphenols have recently been reviewed [103].

Collectively, these findings indicate that Indicaxanthin may act as an antioxidant at concentrations as low as 10 μM, when cells are exposed to certain oxidizing agents. On the other hand, they also clearly highlight the pro-oxidant capacity of high amounts in a lipoperoxide-rich cellular environment. The question that arises is whether in addition to organic oxy-radicals Indicaxanthin scavenges reactive species (ROS/RNS) that are formed when agonists initiate redox-dependent signaling in cells.

#### 6.1.2. Indicaxanthin and Cell Redox Homeostasis: ROS- and RNS-Producing Enzymes and Redox Signaling

Several studies showed that, other than its direct peroxyl radical-scavenging action, Indicaxanthin can prevent the increase of ROS/RNS and changes to the redox balance in various cell lines, when pre- or co-incubated with various agents under simulated patho-physiological conditions [32,33,35,38,39].

Activation/deactivation and/or up-regulation of tissue-specific membrane-bound NADPH oxidases (NOXes) are a general strategy to modify the redox tone of the cells. This is used as a means to convey information and elicit responses in both normal and transformed cells [91,104,105]. NOXes are primarily responsible for oxidative stress associated with noxious stimuli, making these enzymes important pharmacological targets in various disease contexts [106,107]. Dietary-compatible amounts of Indicaxanthin have been shown to prevent ROS production and activation of redox-dependent functional pathways relevant to inflammation and apoptosis, as a result of the inhibition of different NOX isoforms, namely NOX-1 [32] and NOX-4 [33].

Activation of the colonic NOX-1 [108], with production of superoxide anion and -derived oxidants, is considered central to trigger the inflammatory response in the chronic inflammatory bowel disease (IBD) [109,110,111]. Indicaxanthin at 5 to 25 μM dose-dependently abolished ROS elevation in differentiated human Caco-2 cell monolayers submitted to the pro-inflammatory IL-1β cytokine. As a consequence, it also prevented all other ROS-dependent effects: depletion of thiols, NF-κB activation and a number of downstream events leading to over-expression of inflammatory enzymes and release of pro-inflammatory mediators [32]. Interestingly enough, the phytochemical exhibited a “non-antioxidant” behaviour: Indicaxanthin was not consumed and accumulated time-dependently in the colon cells, either in the absence or in the presence of IL-1β, suggesting that it did not remove ROS from the system, rather it affected the production of ROS. NOX-1 activity is dependent on the functional association between cytosolic and membrane subunits [104]. It was observed that Indicaxanthin inhibited the translocation of the cytosolic NOXA-1 activator to the membrane, a critical step in the assembly of a working enzyme complex. By inhibiting NOX-1 activity Indicaxanthin stopped the signalling network triggered by IL-1β and the inflammatory response of colon cells [32].

In other studies [59], 5–80 μM Indicaxanthin down-regulated a number of redox-dependent, pro-inflammatory factors including NOX-1, IL-6, IL-8, COX2, and NOS in Caco-2 cells exposed to a cocktail of cytokines (TNF-α, IL-1β, IFN-γ). The phytochemical, instead, appeared poorly effective in decreasing ROS in the same cells treated with 200 μM H_2_O_2_, while EPR measurements carried out in parallel provided evidence that Indicaxanthin did not possess direct radical scavenging capacity against superoxide [59], in accordance with other observations [32].

Human monocyte/macrophage THP-1 cells underwent oxidative stress-dependent apoptosis when exposed to 7-Ketocholesterol (7-KC) [33]. This highly toxic oxysterol [112] caused an early rise in intracellular ROS due to acute hyper-activation of NOX-4, a NOX isoform whose membrane catalytic subunit is constitutively active to maintain a functional signalling in macrophages [113,114]. The increase of the NOX-4 basal activity was followed by increase of cytosolic calcium, activation of NF-κB, NOX-4 overexpression with sustained gradual rise of ROS and disruption of the redox homeostasis. Added to the medium with 7-KC, 2.5 μM Indicaxanthin prevented the early generation of ROS promoted by 7-KC, thereby preventing the entire sequence of events that stimulate the mitochondrial apoptotic pathway. Notably, Indicaxanthin alone did not affect the NOX-4 basal activity, nor modify the basal level of ROS in the THP-1 cells, suggesting that it did not react with NOX-4-generated reactive species. In contrast, the enzyme appeared to be regulated negatively by the concurrent action of Indicaxanthin and 7-KC: the coexistence of Indicaxanthin and 7-KC completely annulled the basal activity of the enzyme [33].

The models presented in the studies above [32,33,59] reveal interesting issues concerning the potential of Indicaxanthin to prevent oxidative damage in healthy cells: Indicaxanthin (i) does not act as a scavenger of reactive oxygen species; (ii) prevents agonist-induced ROS generation by inhibiting enzymes of the NOX family. The membrane microenvironment has been shown essential in modulating the activity of NOX enzymes [104,115]. A membrane-level activity may be suggested by the physicochemical characteristics of Indicaxanthin: recruiting of effectors, inhibition of the catalytic portion embedded in the membrane, or upstream interference with the IL-1β receptorial machinery [116] are reasonable hypothesis to be tested. More suggestive is that the inhibition of the basal activity of NOX-4, whose membrane catalytic component is not regulated by cytosolic subunits, requires the concurrence of Indicaxanthin and 7-KC [33].

Oxysterols affect cell processes through biophysical mechanisms and alterations of membrane properties [117,118,119]. 7-KC, in particular, has a tendency to destabilize membrane rafts [119,120], which may interfere with rafts-associated functional proteins, including NADPH oxidase [121,122]. The observed hyper-activation of NOX-4 in the THP-1 cells [33] is an example. It is tempting to speculate that co-localization of Indicaxanthin and 7-KC at the membrane of the THP-1 cells may perturb the lipid moiety as to inhibit the NOX-4 catalytic subunit.

Studies in human red blood cells suggest that a joint action of Indicaxanthin and oxysterols can provide anti-oxidative protection by interfering with the activity of ROS-generating enzymes, i.e., RBC-NOX and RBC-NOS. Early results indicated that a mixture of oxysterols enclosing the toxic 7-KC and cholestan-3beta, 5alpha, 6beta-triol (Triol), caused phosphatidyl-serine externalization and cell shrinkage, defined as eryptosis [123], when incubated with freshly isolated erythrocytes [124]. The eryptotic process triggered by the oxysterols strictly depends on the early production of oxidants [124], and can be prevented by 1–5 μM co-incubated Indicaxanthin in a dose-dependent fashion [38]. Indicaxanthin alone did not change the redox environment or affect other parameters of the erythrocyte [38]. Interestingly, 7-KC and Triol concur in determining eryptosis through distinct signalling cascades that activate the membrane RBC-NOX and RBC-NOS, respectively [125]. By building on these evidences [33,38,124,125], one can reasonably postulate that Indicaxanthin and 7-KC and/or Triol may modulate RBC -NOX and/or -NOS activity by acting together. Further investigations, including the selective inhibition of the individual RBC-NOX/-NOS and/or their effectors, could clarify whether Indicaxanthin can exert a distinct and/or specific modulation of the action of these toxic oxysterols.

Other studies show that Indicaxanthin can prevent agent-induced changes to the cell redox homeostasis and activation of oxidant/redox signals capable of inducing (i) inflammation in human umbilical vein endothelial cells (HUVEC) [39,40] and (ii) apoptosis in nervous glial olfactory ensheating cells (OECs) [35].

Mechanical and biochemical stimuli, that involve flowing blood and blood components, concur to activate the endothelium. Among others, oxidized LDL (ox-LDL) and the cytokine TNF-α are considered critical inflammatory agents that can activate the endothelial NOX and generate high level of ROS [126,127,128,129]. Indicaxanthin (5–20 μM) was shown to dose-dependently inhibit the inflammatory response of HUVECs exposed to ox-LDL [39], preventing the increase of intracellular ROS, NF-κB activation and NF-κB-dependent expression of adhesion molecules (ICAM-1, VCAM-1, ELAM-1), while it preserved the activity of the cholesterol efflux system ABC-A1; at the same time, Indicaxanthin did not modify any of the HUVEC basal activities in the absence of ox-LDL [39]. The level of the adhesion molecules at the endothelial cell surface is central in the pathogenesis of the atherosclerotic lesions. Other data show that Indicaxanthin (5 μM), reduced the expression of ICAM-1 induced by treatment of HUVEC with TNF-α by 17% [40]. These studies [39,40] suggest a potential role of the dietary and bioavailable Indicaxanthin to mitigate the atherogenetic effects of ox-LDL and TNF-α in lipid metabolism disturbances and in inflammatory conditions [126,130]. A mechanistic link between Indicaxanthin and inhibition of endothelial NOX isoform(s) may be an important question to investigate.

Indicaxanthin can cross the blood–brain barrier (BBB) [36]. On this basis, its potential to affect ROS production and oxidative stress-dependent cellular responses was recently examined in olfactory ensheating nerve cells [35], a type of glial cells with metabolic characteristics and functions suitable for studying aspects of the Alzheimer’s disease (AD) pathogenesis [131,132]. Exposed to Amyloid-beta (Aβ_1-42_) or its Aβ_25-35_ or Aβ_35-25_ fragments, considered key players in the onset and progression of AD [133,134], OECs over-express trans-glutaminase 2 (TG-2), a multifunctional enzyme that exists in cytosolic (TG-2S) and nuclear (TG-2L) isoforms, the active conformations of which have opposing effects on cell viability, i.e., apoptotic and regenerative, respectively [135,136]. The Aβ-dependent over-expression of TG-2 and its activation in OECs, as well as in other neuronal cells, is associated with ROS production and oxidative stress [137,138,139,140,141,142]. Therefore, the influence of compounds on the Aβ-dependent expression of TG-2 is in the first instance indicative of their ability to counteract the disruption of redox homeostasis.

Under the conditions reported in this study [35], Aβ caused early formation of superoxide anion and ROS, increased expression of total TG-2 with a preponderance of the cytosolic TG-2S isoform, induced gliosis, activation of the mitochondrial apoptotic pathway, caspase-3 cleavage and cell death. Pretreatment (30 min) with Indicaxanthin (100 μM) prevented the Aβ–induced O_2_^•−^and ROS production, and over-expression of total TG-2. It also modulated the conformational transition and subcellular distribution of the enzyme, with increased nuclear TG-2L over cytosolic TG-2S isoform. In doing so, Indicaxanthin promoted the anti-apoptotic (decrease of caspase-3) and cell regenerating (decrease of Vimentin and increase of Nestin and cyclin-D1) effects of the enzyme. Indicaxanthin alone at 100 μM did not change the level of superoxide nor total ROS, nor other activities modified by the exposure to Aβ alone [35].

Considering the role of the Aβ in Alzheimer’s neural degeneration, that a phytochemical able to reach various brain areas [37] can counteract the Aβ apoptotic effects is a remarkable achievement. However, the work raises critical questions while opening up perspectives. In view of the importance and interest in any natural agent that can prevent, mitigate and/or delay the onset and progression of age-related degenerative diseases such as AD, Indicaxanthin may warrant to be studied in other conditions as well. Noteworthy, unlike in RAW264.7 macrophages subjected to LPS [41], such large amounts of Indicaxanthin were not pro-oxidant, rather prevented ROS and O_2_^•−^ production, under the oxidizing conditions in the OECs. This supports the idea that a pro-oxidant behavior of Indicaxathin may not simply result from the abundance and redox capacity of the phytochemical (Section 6.1 and Section 6.1.1), but must perhaps be related to lipid oxidation and its initial activity as a direct antioxidant [25].

The molecular mechanism(s) underlying the activity of Indicaxanthin in OECs remains an interesting question to answer. The complexity of the model, that involves Aβ interactions with OECs membranes and relevant signalling pathways, may offer various points for discussion and further research. Oxidative stress and fine regulation by cytosolic calcium ions are both crucial factors linking Aβ to over-expression, conformational activation and intracellular distribution of TG-2 [137,138,139,140,141,142]. Indicaxanthin could intervene at multiple levels in this context. The production of O_2_^•−^ induced by Aβ, and its prevention by Indicaxanthin, may suggest activation of NOX, in agreement with the effect of Aβ in microglial cells [143,144], and inhibition of the enzyme by Indicaxanthin, as observed in other cell lines [32,33]. In addition, interference by the amphipathic Indicaxanthin for the interaction of the Aβ with OECs membranes [145,146,147] may be considered.

The findings reported through Section 6.1.2 and summarized in Table 3 demonstrate in some cases, while reasonably suggest in others, that Indicaxanthin can prevent the production of ROS, changes in cell redox balance and subsequent redox signaling in various healthy cells exposed to agents that activate NOX enzymes, by inhibiting NOX activity. Although mechanisms at the molecular level are largely unexplored, the amphiphilicity of Indicaxanthin and its interactions with membrane components, e.g., with specialized membrane rafts, may be important. Because of the role of NOXes in maintaining physiological redox homeostasis, in regulating the response in the pathophysiology of many diseases, in pre-malignant transformations [148], and possibly in the cellular aging process itself [149,150], dietary modulators of NOX activity may be critical for life-long protection. Many natural compounds have been described, the inhibitory activity of which relies on various mechanisms of action [151]. Indicaxanthin, shown capable of influencing NOX activity promoted by various agents, at amounts compatible with diet, without affecting the physiological redox status of cells per se, deserves attention and future studies.

### 6.2. Indicaxanthin Activity in Organ Bath

An experimental approach using a mouse ileum preparation showed that, as a component of an aqueous extract of the cactus pear fruit, Indicaxanthin exerted antispasmodic effects on intestinal motility [34]. It is interesting that vitamin C, to the extent of its content in the fruit extract, potentiated the effect of purified Indicaxanthin. Protection of integrity and stability of the phytochemical by ascorbic acid are known in solution under a variety of conditions [152]. Functional interactions between these bioactive dietary components in a complex biological environment may deserve deeper investigation.

On a mechanistic perspective, the myorelaxant effect was independent of neurotransmitter release and ascribed to direct action of Indicaxanthin on the smooth muscle cell, in particular the inhibition of a phosphodiesterase isoform [45]. This caused an increase of intracellular cAMP, a second messenger associated with smooth muscle inhibitory effects [153], including gastrointestinal smooth muscle relaxation [154] (Table 3). Among 11 different isozymes of the PDE family, the PDE-3 and -4 are expressed in the ileal smooth muscle [76,77]. Dynamic interactions between Indicaxanthin and PDE-4 have recently been described by a molecular modelling approach (Section 4). The ability to cross cell membranes [29,32] and reach and accumulate in the cytoplasm could allow Indicaxanthin to target the enzyme. More intriguing though still unexplored possibility is that Indicaxanthin located at the membrane [63,64] would interact with, or cause alteration of the lipid bilayer surrounding the exclusive and large trans-membrane NH-terminal of PDE-3, a segment involved in inhibiting the enzyme activity [76,155].

### 6.3. Indicaxanthin and Oxidative Stress in Tumor Cells

Because cancer is seen as a disorder related to oxidative stress, phytochemicals with antioxidative capacity and a supposedly favourable side effect profile, have been the subject of research to prevent, counteract and treat this disease [156,157]. Chemo-preventive and pro-apoptotic activities and anti-proliferative effects of Indicaxanthin have been shown in several cell lines of different cancers, namely colon [42,46], melanoma [44], and cervical cancer [43], and in a mouse melanoma model as well (Section 7.2.2).

Cancer cells establish and maintain a very different redox tone from that of healthy cells. A sustained imbalance in cell redox homeostasis, caused by elevation of ROS production via mitochondrial dysfunction and NOXes activity [148,158], and/or to a decline of ROS-scavenging capacity, activates oncogenic pathways [159] and is a recognized cause for cancer initiation [160]. Subsequently, cancer cells adapt to high levels of ROS by up-regulation of antioxidant systems, to limit oxidative damage and escape apoptosis. Then, while targeting ROS and ROS signaling with antioxidants is a potential strategy to prevent cancer and/or in the precancerous lesion stage, it may be inappropriate or even detrimental at an advanced stage. Instead, at this point, very high levels of ROS would be required to promote cell death pathways [161,162], or agents that abrogate the antioxidant systems or target unique biochemical features of cancer cells [163,164,165,166]. In this scenario, phytochemicals shown to inhibit proliferation in several tumor cell lines have been associated with several activities: they can act as ROS scavengers; modulate redox-dependent pathways and transcription factors that control gene expression and apoptotic death; activate epigenetic mechanisms; elicit anti-oxidative effects by induction of enzymes involved in antioxidant defence; be pro-oxidant or induce ROS production to kill cancer cells [157,164,167,168]. As for the Indicaxanthin, its antiproliferative effects seemed to be the result of different activities. The in vitro studies have shown that concentrations 5 to 20 times higher than those measured in human plasma after dietary ingestion are required to be effective. The findings are reported in this section, with emphasis on the substantiated mechanisms of action. Based on the known properties of the molecule, additional or alternative mechanistic approaches are also proposed for future research.

Added to proliferating Caco-2 colon adenocarcinoma cells, Indicaxanthin caused a concentration-dependent growth arrest with apoptosis at 48 h, and IC_50_ of 115 μM [42]. The anti-proliferative activity was not associated with changes in the cellular redox balance. Indicaxanthin did not scavenge ROS, nor did it change their intracellular level, nor the level of total thiols [42]. Somewhat intriguingly, this could implicate that Indicaxanthin did not affect the activity of NOX-1, the main source of ROS in colon cancer cells [169], which instead occurred in the differentiated normal-like Caco-2 cells, when submitted to the inflammatory agent IL1-β [32]. Lipid composition and biophysical properties of cancer cell membranes are quite different from those of healthy cells [74,170], it would be meaningful to find out whether and to what extent Indicaxanthin interacts with the membranes of colon cancer or other tumor cells.

Aberrant methylation patterns, i.e., global DNA hypo-methylation contributing to chromosomal instability, but site-specific CpG promoter island hyper-methylation with silencing of tumour suppressor genes, characterize tumor cells, and have been extensively investigated in colorectal cancer [171]. The studies of Naselli et al. [42,46] revealed that Indicaxanthin was able to modulate the epigenome-regulated gene expression by affecting DNA methylation at multiple levels. Treatment of Caco-2 cells with Indicaxanthin (50 μM) increased the global DNA methylation and re-activated the expression of the onco-suppressor p16^INK4a^ gene inducing demethylation of its promoter region [42,46] thus driving cell arrest and apoptosis [172,173,174]. Anti-proliferative effects of Indicaxanthin were also observed in other colon cancer cell lines such as LOVO-1, HCT-116, DLD-1, but not in HT-29, with differences in magnitude of effects, and Caco-2 cells as the most responsive [46]. In addition to p16^INK4a^ Indicaxanthin induced demethylation in the promoters of other onco-suppressor genes (GATA4; ESR1), but left others unchanged (SFRP1; HPP1). The basal methylation seemed to be determinant, being the promoters with the highest methylation levels (SFRP1, HPP1) unaffected [46].

Molecular mechanistic explanations have been attempted. The methylation status of DNA results from the cross-talk between the activity of well characterized methylating enzymes (DNMT), DNMT1, DNMT3A, DNMT3B, and of less known proteins working to transform (TET family enzymes) and remove (MBD4, GADD45A) the methyl groups [175,176]. Indicaxanthin treatment (100 μM) was associated with over-expression of at least one of the DNMTs in all cell lines considered, and of TET2, MBD4, and GADD45A in LOVO1, DLD1 and HCT-116 cells [46]. The remarkably increased expression of DNMT3A (but not DNMT1, nor DMT3B) rationalized the increase in global DNA methylation in Caco-2 cells; however, none of the demethylase genes was affected, in contrast with the GATA4 demethylation and reactivation of the p16^INK4a^ gene [42,46]. Ultimately, a cell-independent procedure showing that Indicaxanthin inhibited the total nuclear DNMT, and an in silico computational modelling analysis revealing a stable binding of the phytochemical at the DNMT1 catalytic site [46], suggest that Indicaxanthin could block the DNA methylation process operating at a gene-specific level [177]. Intracellular uptake and accumulation of Indicaxanthin in Caco-2 cells [32,59], as a necessary precondition for this activity have been observed. Redox regulation and signaling may be upstream of epigenetic modulation [172]; direct inhibition of methylating enzymes could explain the activity of Indicaxanthin in proliferating Caco-2 cells where the phytochemical did not appear to alter the redox tone [42].

Epigenetic modifications, potentially reversible, occur early in the process of carcinogenesis, thus representing a target of choice to prevent malignant transformation in cancers with long precancerous stages such as colorectal cancer. Activity at this level may reveal chemopreventive compounds. Phytochemicals in the diet, which can be taken continuously throughout life, can act as natural controllers in a process at the confluence of redox biology and gene-environment interactions. Importantly, Indicaxanthin selectively acted at the level of modified regulatory mechanisms in the transformed cells and did not affect the viability of non-malignant colon cells, either at the IC_50_ concentration or at twice the concentration [42]. Considering the digestive stability and approximate concentration at the intestine after a cactus pear fruit meal [60,87,178], Indicaxanthin appears to be a chemopreventive component of the diet.

Investigation in melanoma cells [44] provided evidence of a concentration-dependent anti-proliferative effect of Indicaxanthin (50–200 μM) in human A375, cell lines, with growth inhibition going from 20% to 56% at 72 h. A comparable response was observed in murine B16/F10 cells, whereas the inhibitory activity in other cell lines (Sk-Mel-28, MALMF) was remarkably lower. Indicaxanthin did not affect the growth of normal human melanocytes under comparable conditions. With regard to molecular mechanisms, growth arrest and apoptotic death were strictly related to the inhibition NF-κB transcription factor and downstream-regulated events, including the expression of two anti-apoptotic proteins, B cell lymphoma gene-2 (Bcl-2) and FLICE inhibitory protein (c-FLIP).

It may be worth noting that NF-κB is subjected to several controls and activating mechanisms [179] finely tuned by oxidants [180,181,182] and that NADPH oxidases [148,158] and superoxide [183] play a main role in maintaining the aberrant NF-κB activation in cancer cells. Whether Indicaxanthin induced redox changes in the melanoma experimental setup has not been reported, which does not allow confirmation, or refutation, of what has been observed in Caco-2 cells [42], i.e., that Indicaxanthin does not affect NOX activity or ROS level in tumor cells. In addition, the pro-tumorigenic role of NOXes varies with cancer type [148]; in-depth studies on the interaction of NOX with the tumor microenvironment and potential interference by Indicaxanthin should be performed. Other data deserve to be considered. As reported above (Section 4), in silico studies have shown that Indicaxanthin can bind and inhibit the active form of human IKKB, the enzyme responsible for the inactivation of IKΒ-α, the NF-κB inhibitor [79]. Such targeting could offer the advantage of blocking NF-κB directly and would make the action of Indicaxanthin independent of the variety of mechanisms and pathways that activate the NF-κB signaling in a cell- and agent-specific manner [80]. NF-κB has a key role in inflammation, immunity and cancer [184], a reason why new effective and non-toxic compounds to counteract its activity in chronic inflammation, autoimmune diseases, and tumorigenesis are continuously investigated [80]. However, complexities exist for the development of clinically effective inhibitors, as this factor is required to ensure normal immune response and cell survival, i.e., global inhibition of NF-κB function can have serious side effects. Clarifying the aspects and pathways of NF-κB activation and its inhibition by Indicaxanthin in individual diseases will be critical to assessing the potential of the phytochemical as a nontoxic compound to control this factor.

Apart from individual inhibitory effects on cancer cell growth, phytochemicals may exert synergistic effects with other phytochemicals or anticancer drugs [185]. Recent studies in HeLa cells [43] provide further information on the anti-proliferative effect of Indicaxantin and suggest its eventual relevance to enhance chemosensitivity of cisplatin (CDDP). A concentration-dependent antiproliferative activity, related to mitochondrial-dependent apoptosis and arrest of cell cycle, was observed when HeLa cells were treated for 24 h with Indicaxanthin (IC_50_ 149.55 μM) or CDDP (IC_50_ 23.91 μM) alone [43]. Remarkably, when cells were submitted to a combination regimen of Indicaxanthin and CDDP, the Combination Index analysis [186] provided clear evidence of synergism. Moreover, the pre-treatment of HeLa cells with concentrations of Indicaxanthin of nutritional relevance [31], which were ineffective in modifying cell viability per se, potentiated CDDP activity significantly.

Elucidating the mechanism of action of Indicaxanthin in this model, on the one hand would add new knowledge about the potential of the molecule against cancer, but also shed light on the interactions with CDDP, which may serve as a rationale for therapeutic strategies. Apart the formation of adducts with nuclear DNA, the impact of CDDP with mitochondria and mitochondrial genome, followed by ROS generation, has emerged as a cause of cytotoxicity in tumor cells [187,188,189], which was observed in the CDDP-treated HeLa cells. In contrast to CDDP, the apoptotic activity of Indicaxanthin was not related to formation of DCFDA-reactive substances, nor did Indicaxanthin affect key markers of the intrinsic apoptotic pathway, or cell cycle-related proteins or transcription factors. Nevertheless, the CDDP/Indicaxanthin combination resulted in a ROS production and expression of apoptosis-related proteins significantly higher than those observed with CDDP alone.

The importance of elevated production of ROS in the apoptotic action of the combination Indicaxanthin/CDDP was highlighted by the inhibition (over 60%) observed in the presence of NAC [43], however mechanisms through which Indicaxanthin individually promoted apoptosis, or enhanced the prooxidant/apoptotic capacity of CDDP, remain to be clarified. Hypotheses could be made based on previous information. It seems important to emphasize that a substantial anti-proliferative activity of Indicaxanthin in HeLa cells needed concentrations of the order of 10^2^ μM (IC_50_ around 150 μM); on the other hand, nontoxic amounts of the phytochemical (2–10 μM) potentiated the antiproliferative activity of CDDP up to threefold. These findings may suggest distinct activities and possibly molecular mechanisms that complement each other.

Anticancer prodrugs, including phytochemicals, can be activated under high level of oxidants [190], and become pro-oxidant in cancer conditions [191]. Membrane lipids are prone to oxidation in cancer cells, whose environment is rich of ROS [192]. Under these circumstances, pro-oxidant behaviour of Indicaxanthin through activation on lipoperoxides [25,41], can be predicted at the concentrations required to inhibit the HeLa cell growth (50–200 μM). This may eventually promote formation of lipid oxidation breakdown products, including HNE [41], a mediator known to affect cancer cell proliferation [193] and induce apoptosis in HeLa cells [194,195]. Monitoring lipoperoxides and HNE formation and consumption of Indicaxanthin could test this hypothesis.

An activity at the membrane of cancer cells may be considered to explore whether non-cytotoxic Indicaxanthin may facilitate transport/permeation of CDDP. The latter involves interactions of cisplatin with membrane lipids and changes in membrane phase behavior [196], which could be affected by amphipathic Indicaxanthin. Such behavior would be highly relevant to clinical treatment. Facilitating drug entry could effectively decrease the ratio of circulating cisplatin to cellular amount for optimal effect, ultimately reducing the serious side effects of the drug by improving therapeutic efficacy.

The absence of toxicity of elevated amounts of Indicaxanthin observed in various studies [19,42,44,197] suggests safety and potential utility in Combo Therapy to overcome toxicity and drug resistance in cancer treatment [198,199,200,201,202]. CDDP, in particular, has antitumor activity against several types of cancer, which makes it desirable that the activity of their combination be studied in other cancer cells besides HeLa ones, as well as in animal models. In addition, combination of Indicaxanthin with other clinically used anticancer drugs to enhance chemotherapeutic potentiality should be checked.

Table 4 summarizes the effects of Indicaxanthin in tumor cells.

## 7. Indicaxanthin In Vivo: Activity in a Model Organism and Pharmacological Effects in Animal Models

### 7.1. Caenorhabditis Elegans: A New Paradigm for Indicaxanthin?

As a pluricellular model organism, the nematode *Caenorhabditis elegans* is considered suitable for biomedical research, particularly to study mechanisms underlying the aging processes and test the health-promoting and anti-aging potential of molecules. Its relevance in these studies comes from its genome that possesses homologs of about two-thirds of all human disease genes [203,204]. In addition to the wild type, a variety of loss-of-function mutants and transgenic strains allow the identification of signaling pathways and target genes for various compounds. A couple of recent studies report for the first time on the bioactivity and effects of various betalains, including Indicaxanthin, in this nematode [47,48].

Supplied to the TJ375 (*hsp-16.2*:GFP) strain, in a range of concentrations from 10 to 100 μM, Indicaxanthin dose-dependently prevented the juglone-induced, oxidative stress-dependent expression of a green fluorescent protein (GFP), up-regulated by a promoter binding a small 16 kDa heat shock proteins (HSP). The anti-oxidative activity of Indicaxanthin was the highest compared to the natural betanin and synthetic indoline-betacyanin and phenylethylamine-betaxanthin. In addition, wild-type N2 strain nematodes showed increased lifespan and mean survival after being grown for 48 h in liquid culture supplemented with Indicaxanthin, which occurred without accumulation of the phytochemical in the worm [47].

Subsequent studies [48], extended the research to a panel of seventeen natural and synthetic betalain-like molecules, in the attempt to correlate structure of the compounds with the in vitro antiradical activity, protective anti-oxidative activity measured in vivo as juglone-dependent HSP expression, and effects on lifespan. Moreover, the effect on transcription factors and genes known to be involved in *C. elegans* longevity were investigated. On the whole, many of the compounds remarkably reduced the pro-oxidant effect of juglone and extended the lifespan of the worm, however Indicaxanthin performed as the most effective. The pro-longevity effect of Indicaxanthin was mediated through the DAF-16/FOXO transcription factor, with the over-expression of *hsp-16.1* and *lyps-17* as downstream genes pertinent to the oxidative stress resistance and lifespan extension of the worm [205,206]. Transcriptional activity of other relevant factors, such as SKN-1/Nrf-2, was ruled out. Finally, a multivariate analysis using Pearson’s product-moment correlation indicated that the effect of betalains on the lifespan of *C. elegans* can be explained only in part by the reduction of oxidative stress, suggesting that other modulations of signaling pathways must be considered.

These studies, summarized in Table 5, offer various insights to go into and compare the mechanisms underlying the effects of Indicaxanthin in the same organism, under completely different conditions of acute stress [47] or normal growth [48], which can eventually distinguish or reveal signalling pathways that are the same in normal physiology or pathology, and those that are actually novel to pathology. Infact, acute stress changes the state of the environment and possibly the chemistry and reactivity of phytochemicals. Juglone (5-hydroxy-1,4 naphtoquinone), a natural phenolic component of walnut, is a strong redox cycler in vitro (potential E = −93 mV vs. HNE), reacts with oxygen and its reactive species, generating superoxide and H_2_O_2_ and phenol radical intermediates [207]. This implies that in biological settings it may exert either antioxidant or pro-oxidant activities depending on the concentration and on the environmental conditions [208,209]. Much of its bioactivity in normal cells can be related to hormetic-like effects due to pro-oxidant, albeit not toxic amounts, sufficient to promote redox signaling and expression of defense molecules, HSPs being some of these [210].

It is relevant that the heat-shock response can be activated by a range of cellular stressors, including membrane perturbations [211,212], and that juglone can oxidize cellular membranes even at the concentrations that generate signaling (20–50 μM), with formation of lipoperoxides and semiquinone radicals [213]. All this considered, the activity of Indicaxanthin against the pro-oxidant effect of juglone in the TJ375 worm strain could be the reflection of scavenging juglone-generated reactive species, including lipoperoxides. Hypothetically, the lipoperoxyl radical-scavenging activity of Indicaxanthin [25,29] may help prevent the juglone-induced activation of the transcription factor for HSPs, a process induced by oxidants and by lipoperoxidation products such as HNE, among others [214]. Monitoring of the worm’s redox state, lipid peroxide formation, and possibly Indicaxanthin consumption after juglone treatment, and the use of vitamin E as a positive control, could provide some insight into the potential activity of the phytochemical as a true molecular antioxidant in the nematode undergoing acute oxidant stress.

As a next point, molecular mechanism underlying the activation of DAF-16/FOXO transcription factor to extend the lifespan of the *C. elegans* may be hypothesized. An activity of Indicaxanthin as antioxidant is probably not adequate to affect aging under normal growth conditions. Data from Pearson’s analysis point to this direction [48]. Moreover, this conclusion fits with studies showing that longevity of the *C. elegans* can be regulated by mechanisms other than the oxidative stress resistance, and that oxidative damage does not play a significant role in the nematode aging [215,216]. On the contrary, a low level of redox-stress, far from being toxic, is a necessary challenge to trigger transcriptional responses that increase organic defense making cells more resilient to subsequent challenges, which ultimately promotes health and longevity [10,217,218]. The capacity to respond to redox stress tends to be lost in aged worms, but can be improved by pro-oxidant molecules. It is paradigmatic that low amounts (40 μM) of the pro-oxidant juglone, while promoting the expression of heat shock proteins, increases life span in the *C. elegans* [219]. In contrast, the antioxidant resveratrol, while improving stress resistance in a *C. elegans* exposed to 20 μM juglone, did not extend the lifespan of the nematode under normal growth conditions [220]. Can Indicaxanthin create a level of oxidant stress to trigger activation of DAF-16?

Indicaxanthin is chemically stable in healthy cells in the absence of a suitable stimulus, over a wide range of concentration (Section 6.1). Pro-oxidant activity at the expense of cell membrane lipids has been demonstrated under certain conditions [41], however it is not easy to speculate whether equivalent conditions might have occurred in the nematode microenvironment. Oxidatively damaged proteins and lipids accumulate in old vs. young worms, even during normal growth [221]. Hypothetically, the ability of Indicaxanthin to scavenge lipid peroxides could provide a pro-oxidant intermediate [25] and trigger production of a lipid signal to mildly impair mitochondrial function [222], increase the level of ROS and activate DAF-16 [223,224]. Consistent with this hypothesis is the observation that unlike other betalains Indicaxanthin did not accumulate in the *C. elegans* suggesting rapid metabolism/consumption [47]. By virtue of its capacity, Indicaxanthin can perform a dual effect: antioxidant, in the occurrence of acute oxidant stress, but it may contribute to radical formation and pro-oxidant signalling, ultimately participating in defense and lifespan extension. Interestingly Betanin, a good scavenger of superoxide [59] and a good lipoperoxyl radical-scavenger [225], was also rapidly consumed but dit not affect the lifespan of the worm at all [47]. Differently from Indicaxanthin, however, Betanin is not involved in pro-oxidant reactions [225]. The periodic assessment of ROS and stress biomarkers such as lipid oxidation and protein carbonylation products, in the absence or presence of Indicaxanthin, while assessing the timing of consumption of the phytochemical, could contribute to clarify whether and to what extent Indicaxanthin may influence the level of ROS and affect the oxidative damage or not, which might ultimately separate pro-oxidant effects from increased stress resistance in the lifespan of the worm [226]. Evaluating these effects in the presence of antioxidants such as vitamin E and NAC could help shed light on the molecular mechanisms involved. In any case, the wild type worms benefit from the healthful activity of Indicaxanthin, as shown by the significant increase in lifespan, while the molecular mechanisms can be studied in this animal model. In this regard, it might also be interesting to evaluate whether pro-longevity effects of Indicaxanthin can be observed when *C. elegans* is subjected to a stimulating (20–50 μM) or toxic (400 μM) amount of pro-oxidant juglone.

Other ways through which Indicaxanthin might influence longevity could be explored. Physico-chemical characteristics allow activities of Indicaxanthin at various levels, including cell membranes and cytosol, with diverse modes of action including direct binding to proteins (Section 4) and modulation of the activity of membrane-associated enzymes (Section 6.1.2). DAF-16/FOXO requires a redox stress to be activated [223], however its activity is positively or negatively modulated through overlapping signaling, with insulin/IGF-1 being a main regulatory pathway [227,228]. A speculative but exciting topic for a future study would be to investigate whether Indicaxanthin could facilitate DAF-16 activation and nuclear location through direct interactions with regulatory components of the pathways. Finally, NOX enzymes are considered key modulators in aging processes [149,150]. Likewise, ROS-generating dual oxidase [218] appears to play a role in the *C. elegans* longevity and could also be investigated as a site of action of Indicaxanthin.

### 7.2. Pharmacological Activity of Indicaxanthin in Murine Models

The ability of Indicaxanthin to prevent alteration of the endocellular redox state in normal cells submitted to oxidant stress, and modulate signaling pathways in normal, as well as in transformed cells, suggests it could be a promising candidate to exert therapeutic actions. Recent studies with murine models provide evidence that the molecule can exert pharmacological activities in a range of conditions characterized by acute inflammatory patterns or attributable to chronic inflammation-based processes [229,230], including metabolic syndrome linked to obesity, and cancer. In addition, Indicaxanthin can cross the blood–brain barrier, distribute to various brain areas and affect neuronal activity. It should also be noted that it is shown to be effective after oral administration of amounts consistent with a reasonable dietary intake of cactus pear fruit and its bioavailability in humans. The only exception is a melanoma model, where it was effective at five-higher amounts.

#### 7.2.1. Indicaxanthin in a Pleurisy Model

Acute inflammation is the patho-physiological adaptive response to noxious stimuli from bacteria, viruses, toxins, infection, and tissue injury, with the aim of eliminating pathogens and promoting tissue repair and recovery. The carrageenin pleurisy is a reproducible model of acute inflammation, widely considered a gold standard to evaluate anti-inflammatory drugs [231]. Orally administered to rats at 0.5, 1.0, 2.0 μmol/kg, a range consistent with the amount from a reasonable dietary intake of *Opuntia ficus indica* fruits [31], every 8 h up to 40 h, Indicaxanthin antagonized the inflammatory response triggered by injection of λ-carrageenin in a time- and dose-dependent manner [49]. It reduced the exudate volume and the number of leukocytes recruited in the pleural cavity, inhibited the release of PGE2, NO, IL-1β, TNF-α in the exudate, and caused a decrease of IL-1β, TNF-α, iNOS, and COX-2 mRNA, as well as iNOS and COX-2 protein expression in the recruited leukocytes. At a mechanistic level, the effect of Indicaxanthin appeared to be related to a reduced activation of the mediator NF-κB, a pivotal of the inflammatory cascade, responsible for the transcriptional activation of several pro-inflammatory genes [232,233]. The pharmacokinetic profile, assessed after administration of a single oral dose of 2 μmol/kg, allowed the appreciation of the therapeutic plasma concentration of Indicaxanthin. The study showed a maximum mean value of 0.22 μmol/L, with a half-life of 1.15 h [49]. When considering bioavailability in humans [31], these data suggest that Indicaxanthin is an attractive dietary compound to counteract the acute inflammatory process and prevent inflammation-based disorders.

#### 7.2.2. Indicaxantin in a Melanoma Model

Solid evidence links cancer and inflammation [234]. In particular, the causative link between chronic inflammation and melanoma has been widely explored in recent years [235,236,237,238]. It has been shown that reciprocal interactions between melanoma and immune cells in a pro-inflammatory micro-environment provide a source of phenotypic heterogenicity that drives therapy resistance and metastasis [235,239]. Indeed, advanced stages of this skin cancer are characterized by poor prognosis, mainly due to the lack of effective treatments and development of adverse effects of chemotherapy. In this context phytochemicals have been explored and combination therapies evaluated [240]. Antitumoral effects of Indicaxanthin have recently been reported in a mouse model of melanoma [44]. When mice were injected with B16/F10 tumor cells, and co-treated with 3.2 mg/kg (i.e., 10 μmol/kg), three times daily for 14 days, observations 14 days after tumor implantation showed that the phytochemical reduced tumor volume (86%) and weight (83%), and the plasma concentration of the CXCL1, a chemochine associated with the invasive ability of melanoma (42%). Pharmacokinetic parameters were not stated, which prevents an estimation of the therapeutic plasma concentration of Indicaxanthin in this study.

In light of the resistance of melanoma to current therapeutic approaches, the promising chemopreventive potential of Indicaxanthin against this deadly cancer warrants further investigation; in particular, combination therapy studies may open new opportunities.

#### 7.2.3. Indicaxanthin in a Metabolic Syndrome Model

Obesity and related dysmetabolic conditions, peaking in the metabolic syndrome is a serious health problem worldwide [241,242]. Because of a chronic caloric imbalance, that generates adipose tissue hypertrophy and hyperplasia, dysfunctional adipocytes and infiltrating macrophages secrete pro-inflammatory factors, which trigger a sequel of events eventually establishing a state of chronic, systemic, low-grade inflammation. At the same time, the release of pro-inflammatory cytokines and lipotoxic mediators into bloodstream impairs insulin signaling (insulin resistance, IR) and increases adipocyte lipolysis, followed by alteration of the plasmatic lipid pattern, and lipotoxicity. This occurs as ectopic deposition of non-esterified fatty acids (NEFA) in skeletal muscle and hepatic tissue (hepatic steatosis), which further prevents insulin signaling in liver and muscle. The IR state is initially counteracted by increased insulin secretion by pancreatic cells in order to maintain euglycemia. If compensatory insulin secretion fails, beta cells collapse and a diabetic hyperglycemia occurs [243,244,245,246,247,248]. Oxidative stress plays a key role in obesity and is also pivotal for the progression of fat accumulation and insulin resistance [249,250]. All this implies that obesity increases the risk of type-2 diabetes, hypertension and coronary heart disease.

While Western-style eating habits are considered the main culprits in promoting obesity, dietary patterns, foods, and individual bioactive components, including phytochemicals, are now being evaluated to prevent, counteract, or treat obesity-associated disorders. Beneficial effects of Indicaxanthin have recently been observed in a mouse model of high-fat diet (HFD)-induced obesity [50], where the animals progressively develop a pathology similar to human metabolic syndrome, including high increase of body weight and fat mass, hyperglycemia, IR and hepatic steatosis. As an overall impact, a nutritionally relevant dose of Indicaxanthin (1.3 μmol/kg) twice daily for four weeks significantly reduced the food intake, body weight, visceral fat and subcutaneous adipose tissue mass of HFD mice. In addition, the treatment caused a reduction of fasting glycemia and insulinemia, improved glucose (IPGTT) and insulin (ITT) tolerance tests, and restored HOMA index (a marker of insulin resistance). In spite of a restored IR, the Indicaxanthin treatment did not correct abnormalities of the circulating lipid profile (cholesterol, triglycerides) or liver steatosis. Studies reviewing the cellular mechanisms of hepatic insulin resistance and the regulation of hepatic lipid synthesis [246,251], highlight that esterification of preformed fatty acids, the major source of triglycerides in liver, is largely independent on insulin action, which may help explain these data. It was also clearly evident that, unlike other phytochemicals [252,253,254], Indicaxanthin did not directly affect lipid metabolism. Other measurements showed that Indicaxanthin prevented HFD-induced oxidative stress, restoring RONS, MDA and NO levels in liver and adipose tissue, and decreased macrophage infiltration in visceral fat tissue as well as several inflammatory parameters, from TNF-α, CCL-2 and F4-80 gene expression, to p65, p-JNK, COX-2, and i-NOS protein levels in both tissues [50].

Collectively these findings show that, while reducing food intake, body weight and fat mass, Indicaxanthin specifically affects glucose metabolism and corrects IR in a mouse model of metabolic syndrome. Anti-inflammatory activity, inhibition of RONS production and NF-κB/JNK activation in adipose tissue and liver emerge as the main mechanisms by which the phytochemical counteracts obesity and IR. Further studies are necessary to clarify the potential of this nutraceutical as an additive to prevent and treat obesity-related IR in humans and to consider Indicaxanthin as a novel, therapeutic agent for obesity-related disorders. In this context, the observed anoressigenic effect could be an indication to search for additional activities of Indicaxanthin, namely secretion and/or peripheral or brain receptor systems of hormones controlling food intake [255]. The fact that Indicaxanthin can cross the blood–brain barrier and influence neuronal activity (see below) can support this suggestion.

#### 7.2.4. Neuro-Physiological Activity of Indicaxanthin

Probably one of the most interesting aspects of Indicaxanthin bioactivity is its ability to cross the blood–brain barrier and interact with brain cells, exerting neuroprotective effects. As reported in a couple of studies in rats, this occurred after a single administration of 2 μmol/kg body weight, a nutritionally relevant amount corresponding to a dietary consumption of cactus pear fruits in humans [36,37]. Crossing BBB is a very special behavior among phytochemicals, only a few of which (curcumin, resveratrol, tea polyphenols) can have effects on brain cells [256,257,258]. Among all flavonoids, flavanols and anthocyanins are the only ones that accumulate in different regions of the brain, and could be active on memory and learning processes. However, due to limited bioavailability, these molecules are poorly absorbed in the brain, especially the anthocyanins. In contrast, in rats receiving amounts consistent with diet, Indicaxanthin was detectable 60 min after oral administration, and a peak of 20 ± 2.4 ng/g of fresh brain was reached after 2.5 h. The pigment disappeared from the brain at 4 h, according to first-order kinetics with a calculated half-life of 0.82 ± 0.07 h. Completely consistent with the previously reported farmacokinetic profile in rats under comparable conditions [49], a plasma peak of 0.20 μM was observed at 2.5 h, and the molecule disappeared at 4 h. It seems important to highlight that parallel kinetics in plasma and brain suggest simple diffusional mechanisms, in agreement with the high bioavailability, and enabled by Indicaxanthin’s amphiphilicity and affinity for membranes. Taking into account all data and a mean rat blood volume of 16 mL, it can be calculated that the amount of the molecule in the brain at 2.5 h (a time point reflecting an equilibrium of distribution already reached), is approximately 2% of that absorbed.

Once crossed the BBB, Indicaxanthin distributes in rat brain areas and remains unmodified at levels comparable or even greater than those shown by other phytochemicals [37]. Intriguingly, unlike other phytochemicals, the distribution is not homogeneous, but Indicaxanthin gains a specific access to selected brain areas, being present in different amounts in cortex, hippocampus, diencephalon, brainstem, and cerebellum, with a maximum (0.09 ± 0.002) and minimum (0.05 ± 0.002) ng/mg of fresh tissue in cerebellum and cortex, respectively, while it was totally excluded from other areas as the striato-pallidal complex. The peculiar anatomical features of this subcortical region belonging to the basal ganglia and the presence of different fiber bundles could not allow Indicaxanthin accumulation as easily as in the rest of the brain. Molecular structure could also explain its absence from some areas unlike, for example, blueberry anthocyanins, which are capable of entering the striatum [259].

Electrophysiological recordings in rats, applying microiontophoretic techniques, show that Indicaxanthin modulates the bioelectric activity of neurons, suggesting that the phytochemical could influence neuronal plasticity [36,37]. Indeed, when administered in a range between 0.085 ng and 0.34 ng per hippocampal rat neuron, Indicaxanthin modulated the rate of discharge of spontaneously active neurons of the hippocampus in a dose-dependent manner, with reduction of the discharge and related changes of latency and duration of the effect [36]. In line with this, the bioelectric activity of other neurons belonging to different brain areas was also modulated by the phytochemical, mainly with dose-related responses [37]. A predominating inhibitory effect was observed in all the above-mentioned brain areas, as a result of activity at the level of glutamatergic synapses and ability to affect glutamatergic transmission [36]. In support of biochemical and electrophysiological findings, in silico data of molecular modeling show the N-methyl-o-aspartate receptor, the most representative of the glutamate receptor family in hippocampus, as a possible target of Indicaxanthin. Description of the computational studies have been reported [24,36].

Life-long utilization of natural compounds to preserve synaptic plasticity would help prevention of age-related neurological dysfunctions. In this context, dietary Indicaxanthin may be considered to exert beneficial effects by reducing neuronal excitability and act as a functional brake in the context of excitatory circuits, under physiological conditions. Further studies aimed at improving and controlling neuronal delivering could be a premise to envisage this phytochemical as a lead compound for therapeutical perspectives in the field of neurological pathologies, such as neurodegenerative conditions or complex cognitive brain processes, where a dysfunction of neuronal excitability plays a crucial role.

The effects of Indicaxanthin in various animal set-ups modeling pathological circumstances, or focusing physiological conditions, are summarized in Table 6.

## 8. Conclusions

This review paper focuses on the bioactivity of Indicaxanthin, a dietary highly bioavailable betalainic phytochemical with nutraceutical potential. Evidence accumulated so far in healthy, transformed cells and whole organisms suggests health-promoting activities, particularly in counteracting inflammation and regulating mechanisms that control cell growth. The pleiotropic actions of this molecule in vitro and in vivo appear the result of various mechanisms of action and molecular activities. Its redox chemistry and reducing power explain the antioxidant and pro-oxidant activity; the amphiphilicity is essential to enable its activity at cell membranes, including modulation of NOX enzymes, but also the interactions with soluble proteins, so as to justify the molecule’s observed ability to counteract epigenetic modifications, inhibit ileal contractility and inflammation-associated carcinogenesis, or modulate neural bioelectric activity. Many in silico studies suggest that binding with proteins might be an avenue to be explored further with appropriate biological settings. Many observations still require explanation and deserve other studies. New research on the molecular mechanisms related to cell signaling pathways and modulation of gene expression, including suppression of NF-κB oxidative signaling and vitagen activation, could advance our understanding of the possible benefits of Indicaxanthin in maintaining redox homeostasis in normal cells and/or restoring redox homeostasis when a challenge exceeds the capacity of endogenous defense systems. Since Indicaxanthin has shown no deleterious effects in cells and animals, clinical studies could be planned to find useful effective doses for the application of this phytochemical in human health, nutrition, and pharmacology.

## Figures and Tables

**Table 1:** Radical scavenging activity of Indicaxanthin.

Radical	Activity [Ref.]
ABTS^●+ a^	1.76 TE ^b^ [56]; 4.0 TE [48]
ROO^● c^	5.0 TE [30]; 6.0 TE [48]
DPPH^● d^	0.06 TE [30]
NO^● e^	0.28 TE [30]
LOO^● f^	3.6 × 10^5^ M^−1^ s^−1^ [25]
^●^ Hb[Fe^IV^=O] ^g^	660 nMs^−1^ [29]
MPO[Fe^IV^=O]^● h^ MPO[Fe^IV^=O] ^i^ O_2_^●− j^	1.1 × 10^6^ M^−1^ s^−1^ [26] 2.9 × 10^5^ M^−1^ s^−1^ [26] none [59]

^a^ [2,2′-Azinobis(3-ethylbenzothiazoline-6-sulfonic acid)] diammonium salt cation radical; ^b^ Trolox; Equivalents; ^c^ Peroxyl radical generated by thermal decomposition of 2,2′-azobis (2-methyl-propionamidine) dihydrochloride (AAPH); ^d^ 2,2-Diphenyl-1-picrylhydrazyl radical; ^e^ Nitric oxide radical; ^f^ Lipoperoxyl radical; ^g^ Perferryl-hemoglobin; ^h^ Myeloperoxidase-compound I; ^i^ Myelo-peroxidase-compound II; **^j^** superoxide radical.

**Table 2:** Physicochemical properties of Indicaxanthin.

MW ^a^	Log *P* ^b^	Log *D* ^c^	PSA ^d^ (Å^2^)	NPSA ^e^ (Å^2^)	pKa COOH ^f^	Ref.
309 309.3	0.362 −1.12	−7.25	126.92 126.94	161.11	5.0_(2)_, 3.7_(11)_; 2.6_(13)_	[60] [59]

^a^ Molecular weight; ^b^ Octanol/water partition coefficient; ^c^ Octanol/buffer pH 6.0 partition coefficient; ^d^ polar surface area; ^e^ non-polar surface area; ^f^ carbon atom numbers in brackets.

**Table 3:** Bioactivities of Indicaxanthin in cells and tissues.

Cells	Experimental Design	Effect	Key Molecular Mechanisms	Ref.
RBCs (human)	Exposure to cumene hydroperoxide Exposure to toxic oxysterols	Antioxidant Antioxidant Anti-eriptotic	Radical-scavenging 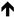 Resistance to oxidative hemolysis 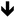 ROS production, glutathione depletion, PGE2 release, and Ca^2+^ entry	[28] [38]
β–Thalassemia RBCs (from patients)	Exposure to cumene hydroperoxide	Antioxidant	Perferryl-Hb reduction 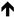 Resistance to oxidative hemolysis 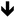 Vit E and GSH depletion 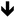 Lipid and hemoglobin oxidation	[29]
HUVEC (human umbilical vein endothelial)	Exposure to oxidized human LDL Exposure to TNFα	Anti-inflammatory Anti-inflammatory	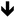 ROS formation 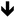 NF-κB transcriptional activity 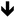 expression of adhesion molecules (ICAM-1; VCAM-1; ELAM-1) Preserved activity of cholesterol efflux system ABC-A1 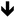 expression of ICAM-1	[39] [40]
RAW 264.7 (murine macrophages)	Exposure to LPS	Anti-inflammatory Pro-oxidant	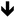 NF-κB activation 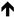 lipid hydroperoxides 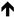 HNE formation Modulation of prostaglandin biosynthetic pathway: 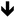 mPGES-1 expression 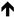 COX2 and MPGDS expression 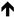 Synthesis of proresolvin cyclopentenone	[41]
Differentiated human Caco-2 (colorectal adenocarcinoma)	Exposure to IL-1β Exposure to a mixture of TNFα, IL-1 β, IFNγ	Anti-inflammatory Anti-inflammatory	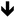 active NOX-1 assembly 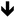 ROS generation 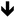 NF-κB activation 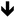 COX2 and NOS expression 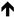 release of IL6, IL8, PGE2, NO 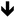 expression of inflammatory IL6, IL8, COX2, NOS, NOX-1 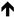 expression of glutamate- cysteine ligase catalytic subunit (GCLC) and glutathione peroxidase-1 (GPX-1)	[32] [59]
THP-1 (human monocyte/macrophages)	exposure to 7-keto-cholesterol	Anti-apoptotic	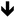 NOX-4 basal activity 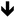 NOX-4 over-expression 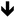 NF-κB activation 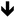 intracellular Ca^2+^ 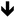 mitochondrial apoptotic pathway	[33]
OECS (olfactory ensheating nerve)	Exposure to amyloid beta	Anti-apoptotic and cell regenerating	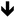 O_2_^•−^ and ROS production 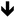 transglutaminase 2 expression 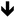 caspase 3 expression 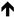 nestin and cyclin-D1	[35]
**Tissues**				
Mouse ileum muscle	Recording of spontaneous or carbachol- or KCl-evoked mechanical activity in organ bath	Spasmolytic	Interference with pathways regulating intracellular Ca^2+^ release 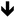 Phosphodiesterase activity 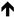 cAMP Vitamin C potentiated the Indicaxanthin effect	[34] [45]

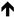
 increase; 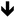 decrease with respect to relevant experiment in the absence of Indicaxanthin.

**Table 4:** Activity of Indicaxanthin in human tumor cells.

Cell Lines	Effect	Key Results	Ref.
Caco-2 (colon adeno-carcinoma)	Anti-proliferative Pro-apoptotic Epigenetic modification	IC_50_ 115 μM Exposure to 115 μM did not affect ROS formation nor total thiols. Exposure to 10–50 μM resulted in: cell cycle arrest at G2/M phase 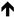 de-methylated p16^INK4a^ gene promoter 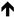 expression and accumulation of the p16^INK4a^ onco-suppressor protein 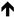 hypo-phospho-RB	[42]
Caco-2 LOVO-1 HCT-116 DLD-1 (colon adeno-carcinoma)	Anti-proliferative (varied with different cell lines) Epigenetic modifications (different sensitivity to Indicaxanthin)	Exposure to 50–100 μM resulted in: 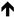 global DNA methylation 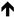 de-methylated onco-suppressor p16^INK4a^, GATA4 ESR1 gene promoters unaffected hypermethylation of SFRP1 and HPP1 gene promoters 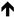 expression of DNA methylating enzymes (DNMTs), enzymes transforming (TET2), and enzymes removing (MBD4, GADD45A) methyl groups 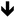 total DNMT activity. In silico computational modeling showed stable binding of Indicaxanthin at the DNMT1 catalytic site.	[46]
HT-29 (colon adeno-carcinoma)	No anti-proliferative effect		[46]
A375 (melanoma)	Anti-proliferative Pro-apoptotic	GI_50_ 100 μM Exposure to 100 μM resulted in: 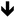 cell growth by 56% after 72 h incubation 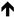 PS externalization 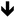 IKB-α degradation 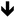 nuclear p65 level 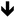 expression of anti-apoptotic Bcl-2 and c-FLIP 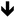 cell invasiveness by 71%	[44]
HeLa (cervical cancer)	Anti-proliferative Pro-apoptotic Synergistic cytotoxic activity in combination with cisplatin (CDDP) Potentiating effect on CDDP toxicity	IC_50_ 149 μM Exposure to 60 μM resulted in: 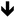 MMP and 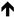 PS externalization arrest of cell cycle at G0/G1 and reduction at G2/M phase unaffected expression of apoptosis- or cell cycle- related proteins unaffected ROS formation or GSH content Indicaxanthin/CDDP (60/10 μM) combination resulted in: 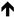 ROS production cell cycle arrest (increase of the cells in G0/G1, absence of cells in G2/M, increase of cells in sub G0/G1 phase) 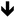 MMP 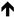 expression of Bax, cyt C, p53, p21^waf1^ and down-regulation of Bcl-2, apoptosis-related proteins NAC decreased the synergistic effects by 60% Non-toxic Indicaxanthin (2–10 μM) potentiated the CDDP toxicity up to three-fold	[43]

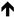
 increase; 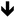 decrease with respect to relevant experiment in the absence of Indicaxanthin.

**Table 5:** Activity of Indicaxanthin in *C. elegans*.

Strain	Effect	Data Evidence	Ref.
TJ375 (*hsp-16.2:GFP*) submitted to pro-oxidant juglone	Antioxidant	Incubation (10–100 μM) for 48 h resulted in: 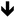 juglone-induced oxidative stress- dependent expression of fluorescent GFP	[47]
Wild type N2	Extension of life-span	Incubation (25 μM) resulted in: 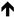 maximum life-span by 3.9 days 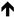 mean survival by 34.34%. Indicaxanthin did not accumulate in the worm	[47]
Wild type N2	Extension of life-span Gene activation	Incubation (25 μM) resulted in: 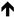 life-span by 16.82%. Incubation (100 μM) resulted in: 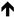 *hsp*-12.1 and *lips*-17 gene expression	[48]
TJ356 (*daf16p:daf-16a/b:GFP + rol-6(su1006*)	Activation of DAF-16/FOXO transcription factor	Incubation (25 μM) resulted in: 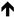 nuclear translocation of DAF-16	[48]
CF1038 (*daf16(mu86*)		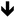 extension of life-span	[48]
LD1 (*ldIs7 [skn-1b/c:GFP + rol-6(su1006*)]		Unaffected SKN/Nrf2 transcription factor No extension of life-span	[48]

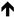
 increase; 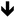 decrease with respect to relevant experiment in the absence of Indicaxanthin.

**Table 6:** Activity of Indicaxanthin in murine models.

	Experimental Set-up	Effect	Data Evidence	Ref.
Rat	λ-Carrageenin-induced pleurisy	Anti-inflammatory	Pre-treatment (0.5–2.0 μmol/kg) orally, every 8 h up to 40 h, resulted in: 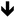 exudate volume 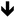 number of leukocytes in pleural cavity 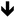 release of PGE2, NO, IL-1β, TNF-α in the exudate 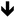 IL-1β, TNF-α, iNOS, COX-2 mRNA and i-NOS and COX2 protein expression in leukocytes 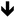 activation of NF-κΒ in leukocytes Peak plasma concentration of Indicaxanthin 0.22 μmol/L, half-life 1.5 h	[49]
Mouse	Cutaneous melanoma	Antiproliferative Impairment of tumor progression	Treatment (3.2 mg/kg) orally, every 8 h for 14 days, resulted in: 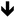 tumor volume (86%) and weight (83%) 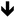 CXCL1 chemochine plasma level	[44]
Mouse	High-fat diet (HFD)-induced obesity (metabolic syndrome model)	Anti-obesity Counteracting of insulin resistance Anti-inflammatory	Treatment (1.3 μmol/kg) orally, every 12 h for 4 weeks, resulted in: 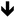 food intake, body weight, visceral fat, subcutaneous adipose tissue mass 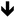 fasting glycemia and insulinemia 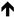 IPGTT and ITT tolerance test restored HOMA index unaffected circulating lipid profile and liver steatosis 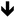 HFD-induced oxidative stress restored RONS, MDA, NO in liver and adipose tissue restored TNF-α, CCL-2, F4-80 gene expression and p65, p-JNK, COX-2, i-NOS protein levels in liver and adipose tissue	[50]
Rat	Pharmacokinetics in brain and neuronal excitability microiontophoretic model	Crossing of BBB Modulatory of neuronal excitability	Treatment (2.0 μmol/kg) orally, single administration, resulted in: accumulation in brain (peak: 20 ng/g fresh tissue at 2.5 h) and disappearance at 4 h with first order kinetics Application (0.085 ng and 0.34 ng per hippocampal neuron) resulted in: 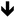 bioelectric activity of hippocampal neurons through glutamatergic synapses	[36]
Rat	Brain distribution and neuronal excitability microiontophoretic model	Neuromodulatory	Treatment (2.0 μmol/kg) orally, single administration, resulted in: selected access to specific brain areas (cerebellum, hippocampus, cortex, diencephalon, brainstem), absence in striatum/ pallidum Application (0.085, 0.17, and 0.34 ng per neuron) resulted in: different effects on neuronal firing rate in different brain areas	[37]

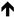
 increase; 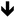 decrease with respect to relevant experiment in the absence of Indicaxanthin
